# A Putative Multicopper Oxidase Encoded by *BAB2_0534* Promotes Melanin-Like Pigment Production and Intracellular Adaptation in *Brucella abortus*

**DOI:** 10.3390/ijms27188105

**Published:** 2026-09-11

**Authors:** Roberto F. Coloma-Rivero, Vicente Arriagada, Leonardo A. Gómez, Raúl Molina, Manuel Flores, Francisco Álvarez, Carolina Delgado-Schneider, Esteban Muñoz-Niklitschek, María Paz-Barrera, Diego Burgos, Claudia Constanzo, Valentina Troncoso, Ángel A. Oñate

**Affiliations:** 1 Laboratory of Molecular Immunology, Department of Microbiology, Faculty of Biological Sciences, Universidad de Concepción, Concepción 4030000, Chile; rcoloma@udec.cl (R.F.C.-R.); viarriagada2021@udec.cl (V.A.); leonardogomez@udec.cl (L.A.G.); ramolina@udec.cl (R.M.); manuelflores@udec.cl (M.F.); fralvarez@udec.cl (F.Á.); 2 Laboratory of Pathological Anatomy and Descriptive Pathophysiology, Faculty of Anatomy, Universidad de Concepción, Concepción 4030000, Chile; caropazdelg@gmail.com (C.D.-S.); estemunoz@gmail.com (E.M.-N.); push2208@gmail.com (M.P.-B.); diego.aburgosb@gmail.com (D.B.); claui.cv@gmail.com (C.C.); 3 Laboratory of Molecular Dynamics of Cancer, Department of Pharmacology, Faculty of Biological Science, Universidad de Concepción, Concepción 4030000, Chile; valetronco@gmail.com

**Keywords:** *Brucella abortus* 2308, multicopper oxidase, laccase, melanin, oxidative stress, reactive oxygen species, intracellular persistence

## Abstract

*Brucella abortus* is an intracellular pathogen that must withstand oxidative stress and other hostile conditions encountered within host cells. Here, we investigated the contribution of *BAB2_0534*, a gene encoding a putative multicopper oxidase, to bacterial adaptation and persistence. Deletion of *BAB2_0534* impaired pigment production in the presence of the melanin precursors L-DOPA and homogentisic acid, supporting a role for *BAB2_0534* in phenolic substrate oxidation and melanin-like pigment biosynthesis. The mutant also exhibited increased susceptibility to oxidative stress and reduced survival with macrophages, indicating that *BAB2_0534* contribute to intracellular adaptation. In vivo, mice infected with the Δ*BAB2_0534* mutant showed significantly reduced splenic bacterial burdens compared with animals infected with the wild-type or complemented strains, demonstrating impaired persistence during infection. Consistently, Fontana–Masson-positive pigment was detected in tissues from wild-type-infected animals but was markedly reduced following infection with the mutant. Collectively, these findings support a model in which *BAB2_0534*-associated multicopper oxidase activity and pigment production contribute to oxidative stress resistance and intracellular persistence of *B. abortus*. This study characterizes *BAB2_0534* as a previously unrecognized component of the adaptive mechanisms underlying *Brucella* persistence.

## 1. Introduction

*Brucella abortus* is a Gram-negative facultative intracellular pathogen and the principal causative agent of bovine brucellosis, a globally distributed zoonosis that imposes substantial economic losses on livestock production and remains an important public health concern [1,2,3]. The success of *Brucella* as an intracellular pathogen relies on its ability to survive and replicate within professional phagocytes, where it establishes a specialized replicative niche and evades host bactericidal responses [4,5,6]. This remarkable intracellular lifestyle depends on the coordinated activity of multiple virulence determinants that regulate intracellular trafficking, immune evasion, metabolic remodeling, and stress adaptation, ultimately enabling long-term persistence and chronic infection [7,8]. Although considerable progress has been made in identifying the molecular components involved in these processes, the mechanisms that integrate bacterial responses to the diverse physicochemical stresses encountered within host cells remain incompletely understood.

During intracellular infection, *Brucella* is exposed to a complex combination of antimicrobial defenses, including reactive oxygen species (ROS), reactive nitrogen species (RNS), nutritional immunity, acidic pH, and transition-metal intoxication, all of which compromise bacterial physiology by disrupting redox homeostasis, protein function, and essential metabolic pathways [9,10]. Among these stresses, copper has emerged as a central component of the innate antimicrobial response. Activated macrophages actively accumulate copper within phagosomal compartments, where the reduced Cu(I) form promotes oxidative damage, protein mismetallation, and metabolic dysfunction [11,12]. Consequently, successful intracellular pathogens require mechanisms that not only detoxify excess intracellular copper but also coordinate metal homeostasis with oxidative stress resistance to preserve cellular integrity during infection [13]. Although *Brucella* possesses several antioxidant systems that contribute to resistance against oxidative damage [14,15], whether additional mechanisms function at the interface between copper homeostasis, redox regulation, and intracellular adaptation remains largely unknown.

Bacterial multicopper oxidases (MCOs) represent an attractive class of enzymes that could contribute to intracellular adaptation by linking copper homeostasis with oxidative stress resistance [16]. These enzymes catalyze the oxidation of the highly reactive Cu(I) ion into the less toxic Cu (II) form, thereby reducing copper-associated toxicity while maintaining intracellular metal balance [17,18,19]. However, the biological functions of bacterial MCOs extend beyond copper detoxification. Increasing evidence indicates that these enzymes can oxidize a wide range of phenolic compounds and participate in pigment biosynthesis, extracellular redox processes, and environmental stress adaptation [20,21]. Among the biological outcomes associated with MCO activity, melanin production is particularly intriguing because this polymer provides multiple protective functions, including reactive oxygen species scavenging, transition-metal chelation, and enhanced resistance to host-derived antimicrobial stresses [22]. In fungal pathogens, laccase-dependent melanin biosynthesis is a well-established virulence mechanism that promotes survival under oxidative stress and contributes to persistence within the host environment [23,24]. In contrast, whether melanin production contributes to the pathogenic lifestyle of intracellular bacterial pathogens remains largely unexplored [22,25].

Genome annotation of *B. abortus* 2308 identifies the open reading frame *BAB2_0534* (BAB_RS28900) as encoding a predicted multicopper oxidase belonging to the bacterial CueO family [26]. The predicted protein contains conserved copper-binding motifs characteristic of bacterial MCOs and exhibits significant sequence similarity to the experimentally characterized multicopper oxidase BmcO from *Brucella melitensis*, suggesting a conserved role in copper-dependent enzymatic processes [27]. Nevertheless, the physiological function of *BAB2_0534* in *B. abortus* has not been experimentally characterized. It remains unknown whether this protein functions exclusively as a copper homeostasis factor or whether it contributes to broader adaptive responses required for intracellular survival.

Understanding the role of *BAB2_0534* may provide new insights into how *Brucella* coordinates multiple stress responses during infection. Within macrophages, copper intoxication, oxidative stress, and nutrient limitation occur simultaneously, creating a selective pressure for bacterial factors capable of integrating these environmental challenges rather than responding to them independently [28,29]. Therefore, melanin biosynthesis associated with MCO activity may represent not merely a secondary enzymatic consequence but a functional adaptation that enhances bacterial resilience by limiting oxidative damage, maintaining redox equilibrium, and modulating metal availability within the intracellular niche [22,23]. Demonstrating such a mechanism would redefine the role of bacterial MCOs from enzymes primarily involved in copper detoxification to multifunctional determinants that contribute to pathogen adaptation and persistence.

Based on these observations, we hypothesized that *BAB2_0534*, a protein predicted to belong to the multicopper oxidase family, may link copper homeostasis, laccase-like activity, and melanin biosynthesis as part of an adaptive response to intracellular stress in *B. abortus*. To test this hypothesis, we characterized the biological function of *BAB2_0534* and investigated its contribution to copper tolerance, melanin production, oxidative stress resistance, intracellular survival, and bacterial virulence. These studies characterize the physiological contribution of the previously uncharacterized *BAB2_0534* protein to bacterial adaptation and persistence in *B. abortus*.

## 2. Results

### 2.1. Genomic and Structural Analyses Identify BAB2_0534 as a Putative Multicopper Oxidase Within a Conserved Metal Homeostasis-Associated Locus

**Figure 1:**
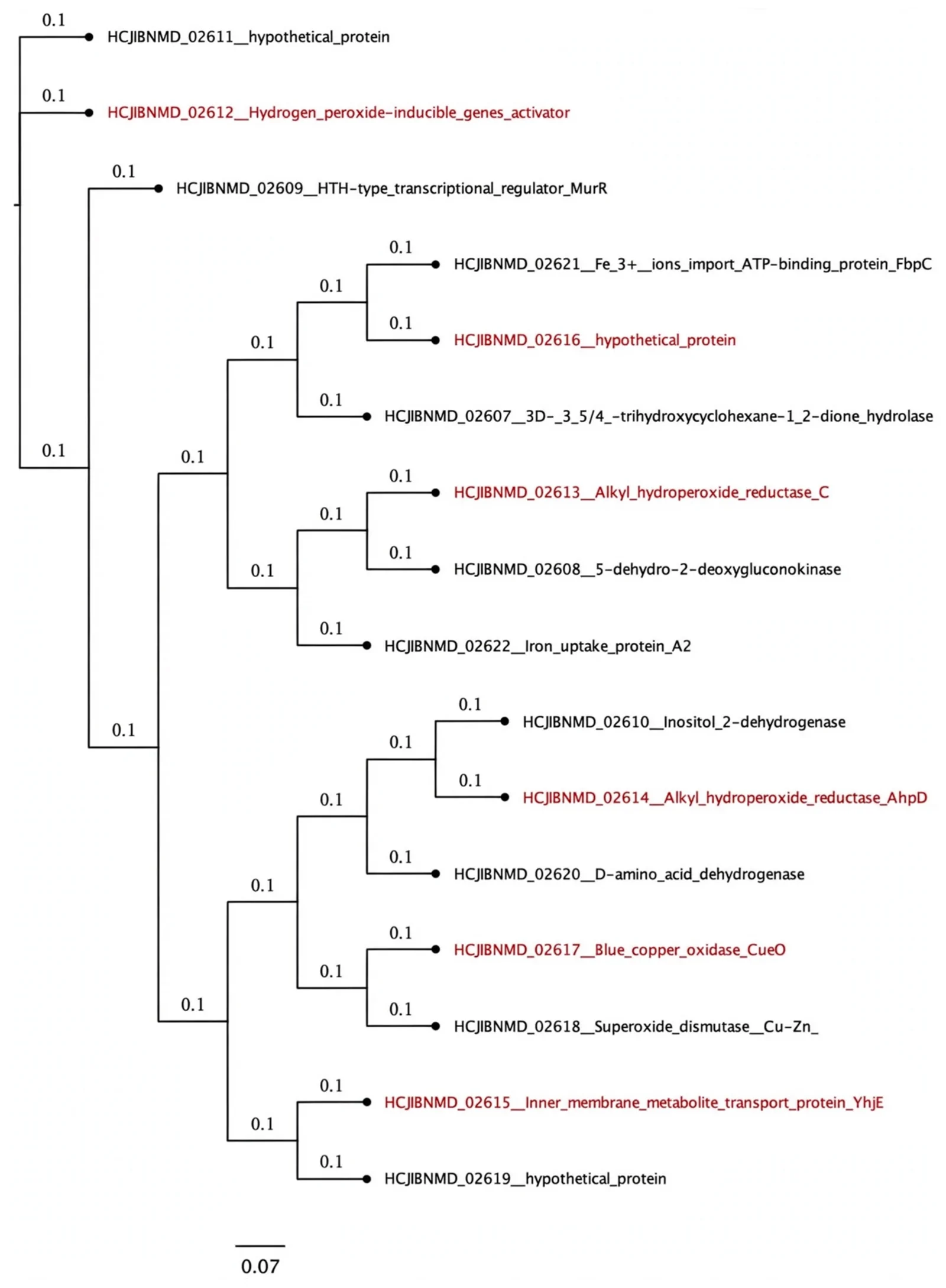
Sequence similarity relationships among proteins encoded within the *BAB2_0534* genomic neighborhood. Tree generated from the predicted amino acid sequences encoded by the BAB2_0530–BAB2_0535 genomic region and five flanking genes upstream and downstream in *B. abortus* 2308. The analysis illustrates the sequence relationships among proteins encoded within this stress-associated locus. Genes involved in oxidative stress defense, metal homeostasis, and the putative multicopper oxidase *BAB2_0534* (HCJIBNMD_02617; annotated as Blue_copper_oxidase_CueO) are highlighted in red.

Genomic analysis of *B. abortus* 2308 identified the BAB2_0530–BAB2_0535 region (positions 143,486–150,165) as a conserved cluster of consecutive genes (Table 1). Phylogenetic analysis revealed a short evolutionary distance among the corresponding sequences, supporting strong conservation of this genomic region (Figure 1). Operon prediction further identified a putative operon encompassing BAB2_0530 and BAB2_0531 (Figure S1).

**Figure 2:**
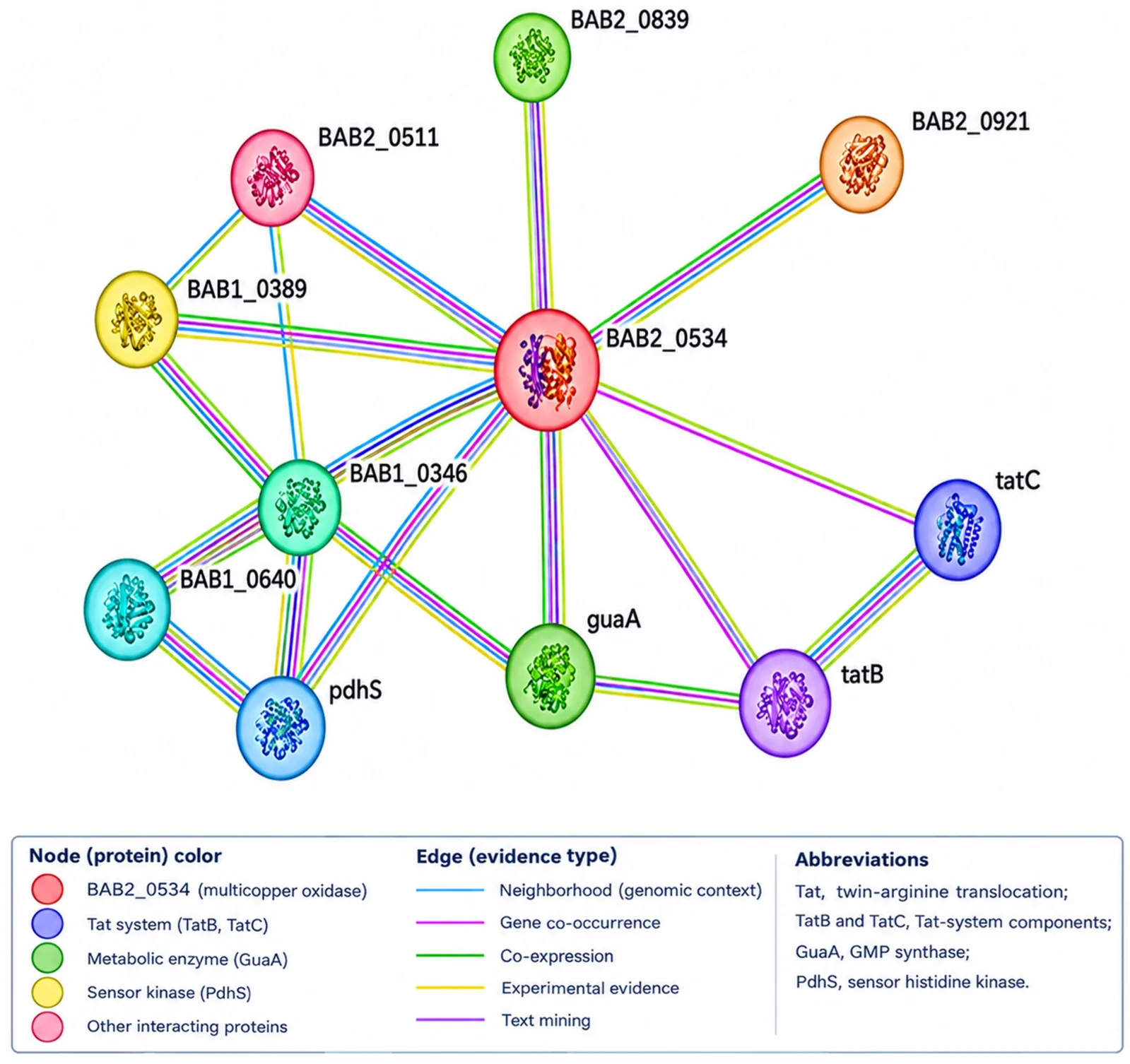
Predicted functional association network of *BAB2_0534*. The functional network was generated using the STRING database, with *BAB2_0534* (red central node) representing the putative multicopper oxidase. The network predicts functional associations with components of the twin-arginine translocation (Tat) system (TatB and TatC), metabolic enzymes, and other proteins potentially involved in cellular adaptation and metal homeostasis. Nodes represent proteins, whereas edges indicate predicted functional associations based on evidence integrated by STRING, including genomic context, gene co-occurrence, co-expression, and available experimental evidence. Abbreviations: Tat, twin-arginine translocation; TatB and TatC, Tat-system components; GuaA, GMP synthase; PdhS, sensor histidine kinase.

Inspection of the surrounding genomic context showed that this locus is flanked by genes involved in oxidative stress defense and metal homeostasis, including a Cu-Zn superoxide dismutase (HCJIBNMD_02618), the ferric iron ATP-binding transporter FbpC (HCJIBNMD_02621), and the iron uptake protein A2 (HCJIBNMD_02622). Consistent with this genomic context, STRING analysis predicted functional associations between *BAB2_0534* and at least ten proteins, notably components of the twin-arginine translocation (Tat) system (TatB and TatC), plastocyanin, the iron permease FTR1 (BAB2_0838), as well as enzymes involved in central metabolism, including glutamate synthase and acetolactate synthase (Figure 2).

**Figure 3:**
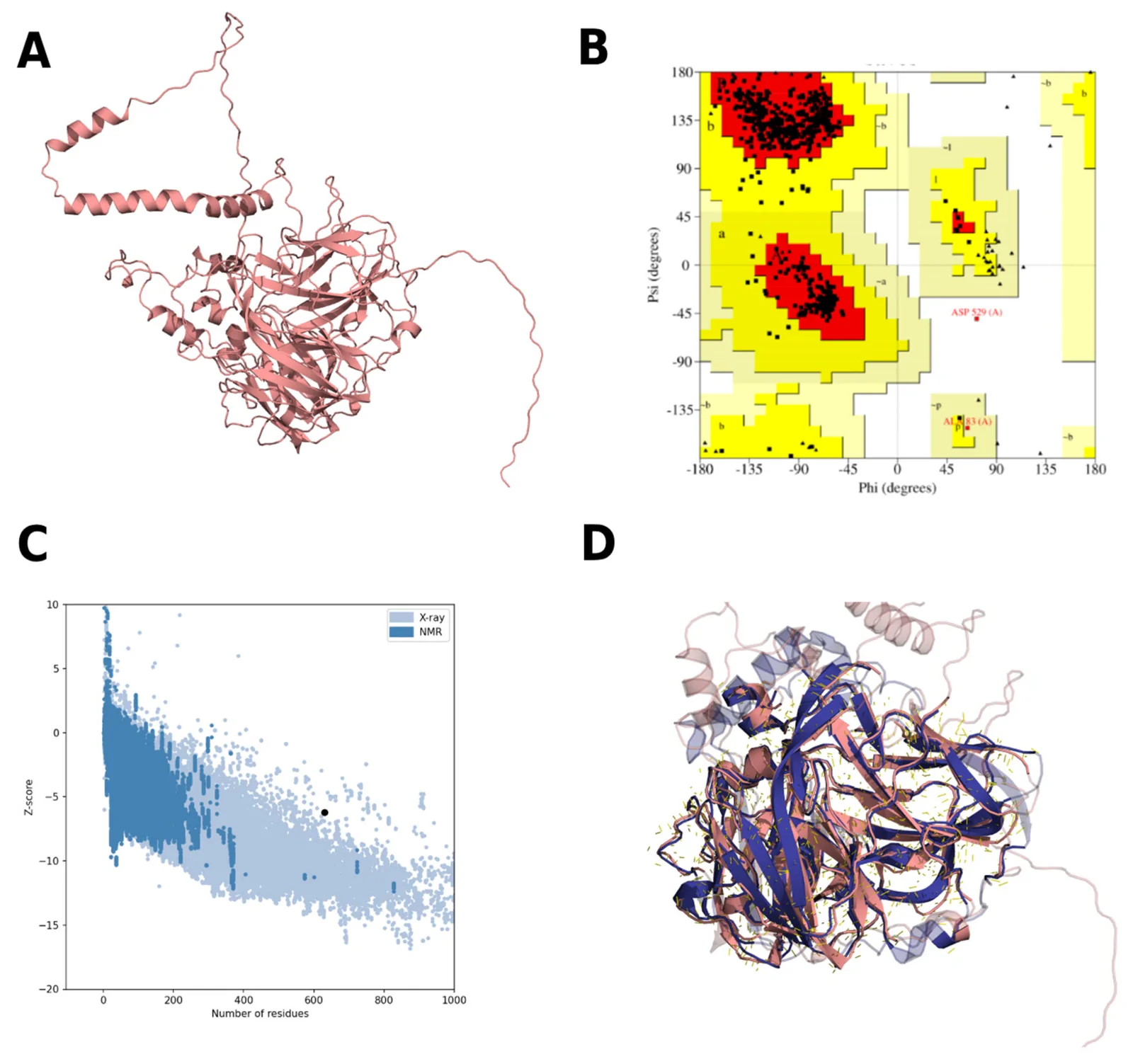
Structural characterization of the predicted *BAB2_0534* multicopper oxidase. (**A**) Predicted three-dimensional structure of *BAB2_0534* shown in cartoon representation. (**B**) Ramachandran plot assessing the stereochemical quality of the predicted model, with 99.4% of residues located in favored or additionally allowed regions. Residues located outside these regions correspond to disallowed conformations. The letters a, b, l, and p denote the characteristic conformational regions represented in the plot. (**C**) ProSA-web analysis evaluating the overall quality of the predicted structure, showing a Z-score of −6.22 (black dot). (**D**) Structural superimposition of the predicted *BAB2_0534* model (pink) and the crystal structure of the multicopper oxidase CueO from *E. coli* (blue), revealing a high degree of structural conservation (RMSD = 0.814 Å over 1965 aligned atoms).

*BAB2_0534* is predicted to encode a hypothetical 631-amino acid protein with a molecular weight of 68.4 kDa and a theoretical isoelectric point (pI) of 6.04. In silico analyses predicted that the protein is stable (instability index = 38.97), moderately hydrophilic (GRAVY = −0.136), and potentially thermostable (aliphatic index = 82.65). Subcellular localization analysis suggested that *BAB2_0534* is predominantly localized in the cytoplasm and periplasm. The predicted structural model showed excellent stereochemical quality, with 99.4% of residues located in allowed regions and a ProSA Z-score of −6.22 (Figure 3B,C). Structural comparison with the well-characterized multicopper oxidase CueO from *Escherichia coli* revealed a high degree of structural similarity, with an RMSD of 0.814 Å over 1965 aligned atoms (Figure 3D), supporting the annotation of *BAB2_0534* as a putative multicopper oxidase.

### 2.2. BAB2_0534 Contributes to Optimal In Vitro Growth of B. abortus

**Figure 4:**
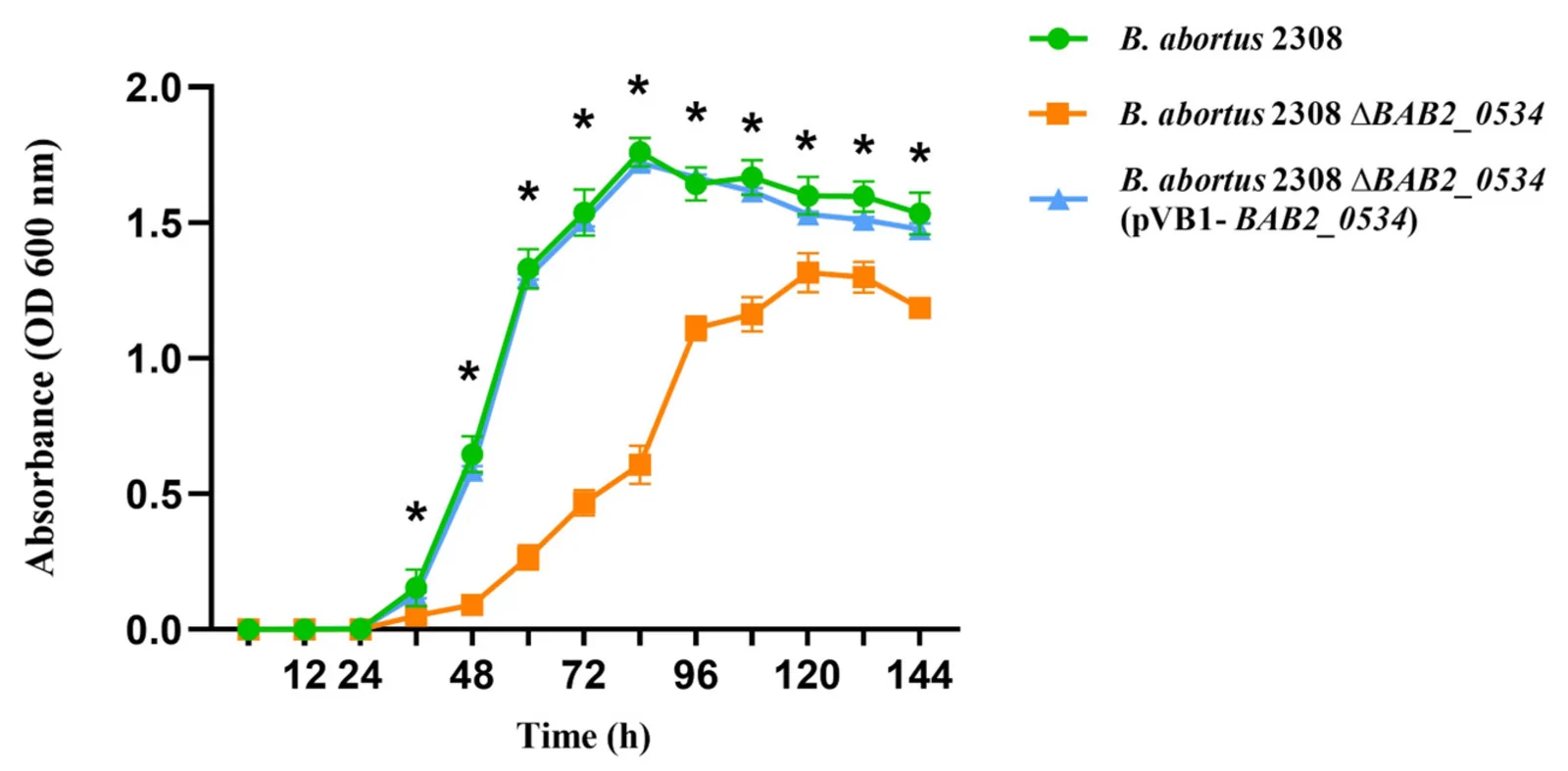
Deletion of *BAB2_0534* impairs the in vitro growth of *B. abortus*. Growth kinetics of wild-type *B. abortus 2308* (green circles), the isogenic Δ*BAB2_0534* mutant (orange squares), and the complemented strain Δ*BAB2_0534*(pVB1-*BAB2_0534*) (blue triangles) cultured in Brucella broth. Data are presented as the mean ± standard deviation (SD) from three independent biological replicates. Statistical significance was determined by two-way ANOVA followed by Tukey’s multiple-comparisons test. * indicates *p* < 0.05 for comparisons between the Δ*BAB2_0534* mutant and the wild-type strain.

The effect of *BAB2_0534* deletion on bacterial fitness was evaluated by monitoring growth kinetics in Brucella broth. The Δ*BAB2_0534* mutant displayed delayed growth, entering the exponential phase only after 48 h, whereas the wild-type strain reached this phase substantially earlier. In addition, the mutant exhibited a significantly lower exponential growth rate. Consistent with this phenotype, the wild-type strain reached stationary phase after 72 h, while the mutant did not do so until 96 h, demonstrating a marked in vitro growth defect. In contrast, the growth kinetics of the complemented strain [Δ*BAB2_0534*(pBV1-*BAB2_0534*)] were indistinguishable from those of the wild-type strain, indicating that the impaired growth phenotype was specifically attributable to the absence of *BAB2_0534* (Figure 4).

### 2.3. BAB2_0534 Is Required for Eumelanin-Like Pigment Production In Vitro

**Figure 5:**
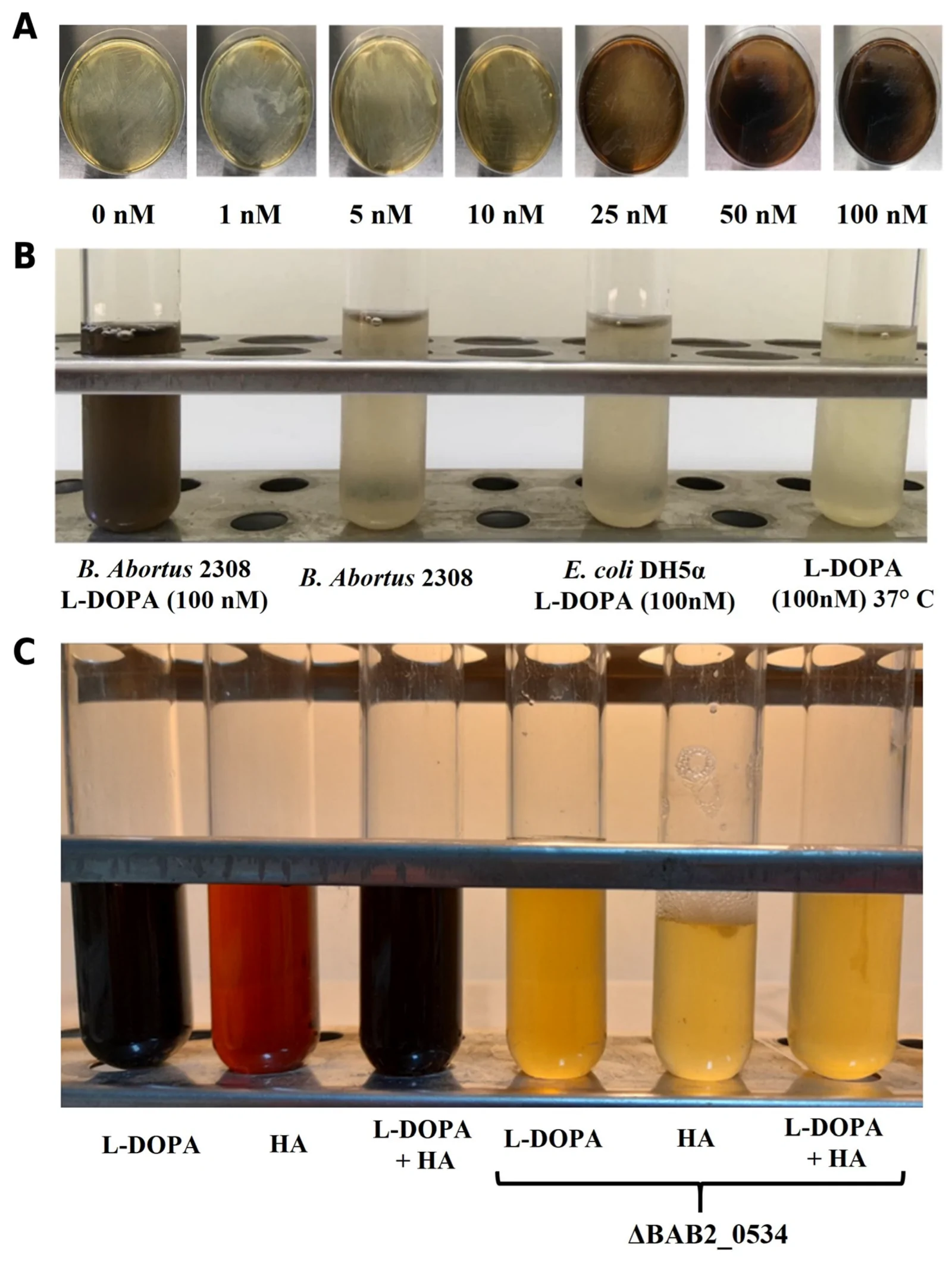
*BAB2_0534* is required for extracellular melanin-like pigment production in *B. abortus*. (**A**) Dose-dependent production of a dark, diffusible melanin-like pigment by wild-type *B. abortus* 2308 cultured on Brucella agar supplemented with increasing concentrations of L-DOPA (0–100 nM). Bacterial colonies remained unpigmented throughout the assay, whereas the pigment accumulated in the surrounding medium. (**B**) Melanin-like pigment production in liquid culture after 72 h of incubation. A dark-brown pigment accumulated in the culture supernatant of wild-type *B. abortus* 2308 grown in the presence of 100 nM L-DOPA, whereas no pigmentation was observed in the wild-type culture without L-DOPA, in *E. coli* DH5α supplemented with L-DOPA (negative biological control) or in uninoculated L-DOPA-containing medium incubated at 37 °C (auto-oxidation control). (**C**) Comparison of melanin-like pigment production by wild-type, Δ*BAB2_0534*, and complemented strains cultured in brucella broth supplemented with L-DOPA, homogentisic acid (HA), or a combination of both substrates.

To investigate the role of *BAB2_0534* in melanin-like pigment production*, B. abortus* 2308 was cultured in media supplemented with the melanin precursors L-DOPA and homogentisic acid (HA). Pigment production increased with increasing L-DOPA concentrations, becoming clearly detectable at 25 nM and progressively increasing at 50 and 100 nM (Figure 5A). At 100 nM L-DOPA, a dark, diffusible pigment accumulated in the culture medium, whereas the bacterial biomass remained whitish. No comparable discoloration was observed in *E. coli* DH5α cultured with L-DOPA or in L-DOPA incubated at 37 °C in the absence of bacteria, supporting a bacteria-dependent process (Figure 5B). Furthermore, *B. abortus* 2308 produced dark pigmentation in the presence of L-DOPA or L-DOPA plus HA, whereas HA alone produced a reddish pigment. In contrast, the Δ*BAB2_0534* mutant failed to produce the dark pigmentation observed in the wild-type strain under these conditions, instead retaining a yellowish appearance (Figure 5C). Together, these results indicate that *BAB2_0534* is required for the formation of the dark, melanin-like extracellular pigment produced by *B. abortus* 2308 in the presence of L-DOPA and contributes to pigment formation in the presence of HA.

**Figure 6:**
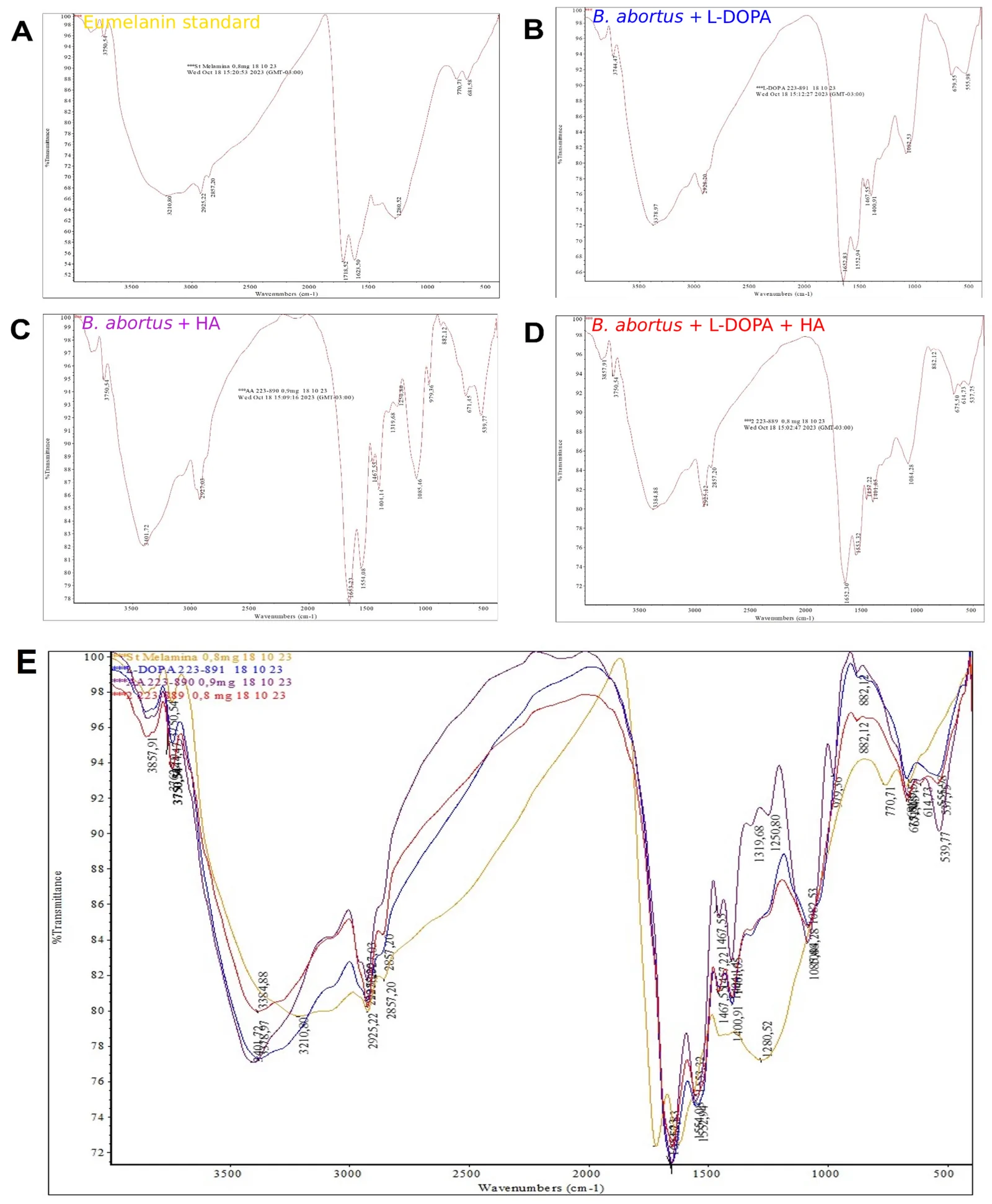
Fourier-transform infrared (FTIR) spectroscopy supports the eumelanin-like nature of the *BAB2_0534*-associated extracellular pigments. Representative FTIR transmission spectra of (**A**) a commercial eumelanin standard and purified bacterial pigments extracted from *B. abortus* 2308 cultured in the presence of (**B**) L-DOPA, (**C**) homogentisic acid (HA), and (**D**) L-DOPA combined with HA. (**E**) Superimposition of FTIR spectra comparing the commercial eumelanin standard (yellow line) with bacterial pigments obtained from L-DOPA (blue line), HA (purple line), and L-DOPA + HA (dark red line). Abbreviations: FTIR, Fourier-transform infrared spectroscopy; L-DOPA, L-3,4-dihydroxyphenylalanine; HA, homogentisic acid. *** It represents the different experimental treatments.

To characterize the extracellular pigment, precipitates recovered from wild-type culture supernatants were analyzed by Fourier-transform infrared (FTIR) spectroscopy. The infrared spectrum of a commercial melanin standard displayed the characteristic banding pattern of eumelanin (Figure 6A). Likewise, pigments purified from cultures supplemented with L-DOPA (Figure 6B), HA (Figure 6C), or L-DOPA plus HA (Figure 6D) exhibited FTIR spectra highly consistent with a eumelanin-like banding pattern. A notable difference was observed at 1281.52 cm^−1^, where the peak present in the commercial standard (Figure 6E) was absent or altered in the bacterial pigments, a feature previously reported for soluble bacterial melanins. Collectively, these findings indicate that *BAB2_0534* is required for the production of extracellular melanin-like pigments with FTIR signatures consistent with eumelanin.

### 2.4. BAB2_0534 Promotes Intracellular Survival and Modulates Macrophage Responses

**Figure 7:**
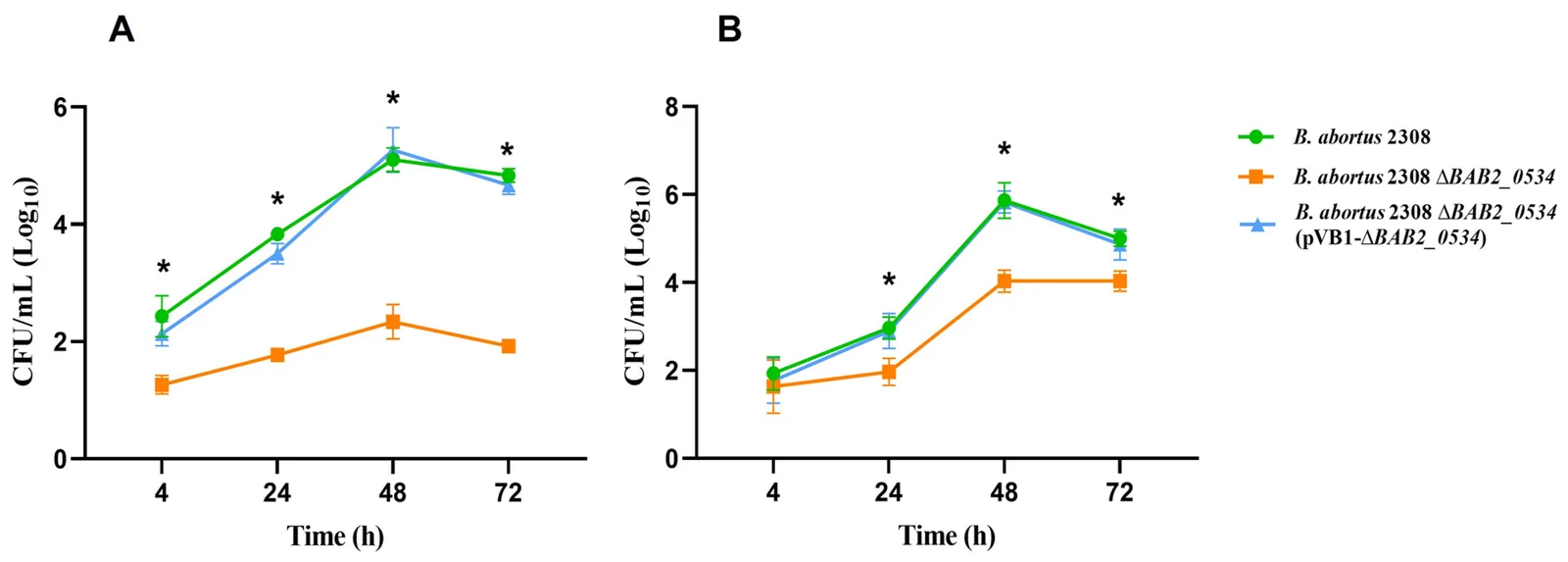
*BAB2_0534* contributes to intracellular survival of *B. abortus* in professional and non-professional phagocytic cells. Intracellular replication kinetics of wild-type *B. abortus* 2308 (green circles), the Δ*BAB2_0534* mutant (orange squares), and the complemented strain Δ*BAB2_0534*(pVB1-*BAB2_0534*) (blue triangles) in (**A**) murine RAW264.7 macrophages and (**B**) human HeLa epithelial cells. Cells were infected at a multiplicity of infection (MOI) of 500:1, and viable intracellular bacteria (log_10_ CFU/mL) were quantified at 4, 24, 48, and 72 h post-infection. * Data represent the mean ± standard deviation (SD) from three independent biological replicates (*n* = 3). Statistical significance was determined by two-way ANOVA followed by Tukey’s multiple comparisons test (*p* < 0.05).

To evaluate the role of *BAB2_0534* during cellular infection, intracellular survival was assessed in RAW264.7 macrophages and HeLa epithelial cells (MOI 500:1). At 4 h post-infection (p.i.), all *Brucella* strains exhibited similar intracellular CFU counts in macrophages (Figure 7A). However, between 24 and 72 h p.i., the Δ*BAB2_0534* mutant exhibited a significant reduction in survival (~2 log_10_ CFU/mL) compared to the wild-type and complemented strains (*p* < 0.05). A similar defect was observed in HeLa cells: while intracellular counts were comparable at 4 h (2.3 log_10_ CFU/mL) and 24 h (2.9 log_10_ CFU/mL), the mutant displayed approximately 2.2 log_10_ fewer CFU/mL than the wild-type and complemented strains between 48 and 72 h p.i. (Figure 7B).

**Figure 8:**
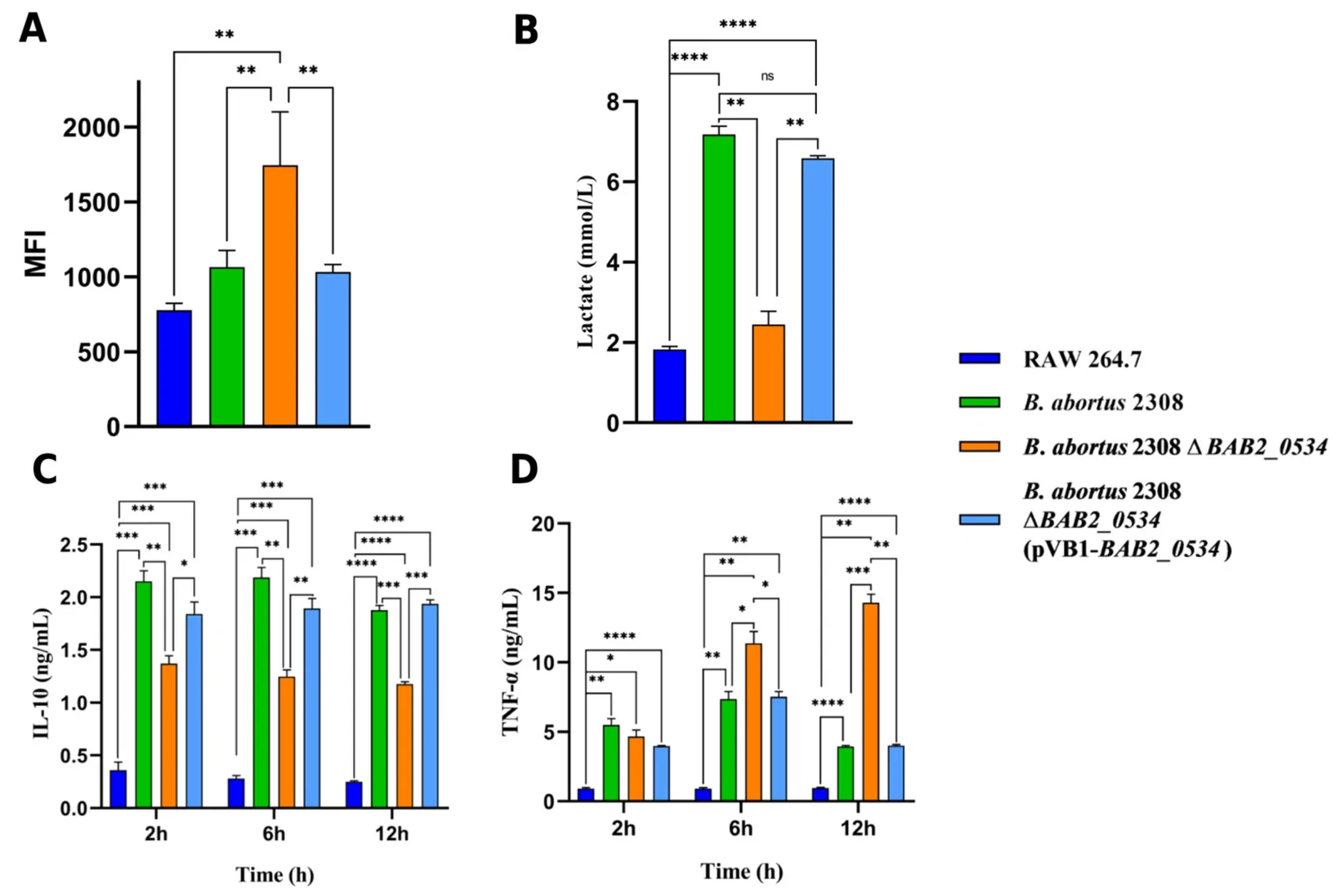
*BAB2_0534* reduces macrophage oxidative burst and modulates inflammatory responses during intracellular infection. RAW 264.7 macrophages were mock-infected (dark blue) or infected with wild-type *B. abortus* 2308 (green), the Δ*BAB2_0534* mutant (orange), or the complemented strain Δ*BAB2_0534*(pVB1-*BAB2_0534*) (light blue). (**A**) Intracellular reactive oxygen species (ROS) production measured as mean fluorescence intensity (MFI). (**B**) Intracellular lactate concentrations (mmol/L). (**C**) Secretion levels of the immunoregulatory cytokine IL-10 and (**D**) the pro-inflammatory cytokine TNF-α (ng/mL) quantified at 2, 6, and 12 h post-infection. Data represent the mean ± standard deviation (SD) from three independent experiments. Statistical significance was assessed by one-way or two-way ANOVA (as appropriate) followed by Tukey’s multiple comparisons test (* *p* < 0.05, ** *p* < 0.01, *** *p* < 0.001, **** *p* < 0.0001; ns, not significant).

The impact of *BAB2_0534* on host cell physiology was further investigated in RAW264.7 macrophages. Quantification of reactive oxygen species (ROS) revealed that macrophages infected with the Δ*BAB2_0534* mutant produced significantly higher ROS levels (*p* < 0.01) compared to all other experimental groups. In contrast, ROS production in cells infected with either the wild-type or the complemented strain remained comparable to that of uninfected cells (Figure 8A). Furthermore, the influence of *BAB2_0534* on host cell metabolism was assessed by quantifying intracellular lactate levels. Infection with wild-type *B. abortus* 2308 or the complemented strain induced a highly significant increase in lactate production compared to uninfected control cells (*p* < 0.0001). Conversely, macrophages infected with the Δ*BAB2_0534* mutant failed to induce the increase in lactate production observed in macrophages infected with the wild-type or complemented strains, remaining at levels comparable to uninfected controls. Consequently, intracellular lactate concentrations in mutant-infected cells were significantly lower than those observed in cells infected with either the wild-type or complemented strains (*p* < 0.01). No significant differences were detected between the wild-type and complemented groups, confirming full restoration of the metabolic phenotype (Figure 8B). Finally, cytokine analysis revealed that the Δ*BAB2_0534* mutant induced significantly lower IL-10 secretion at all evaluated time points relative to the wild-type strain (Figure 8C). Conversely, the mutant strain triggered a markedly higher production of the pro-inflammatory cytokine TNF-α at 6 and 12 h p.i. Notably, TNF-α secretion in mutant-infected macrophages increased progressively over time, and the statistical divergence between the mutant and wild-type groups became more pronounced at later stages, with the strongest statistical difference observed at 12 h p.i. (*p* < 0.001) (Figure 8D). Collectively, these findings indicate that *BAB2_0534* contributes to intracellular persistence and is associated with reduced oxidative and inflammatory activation of infected macrophages, supporting its proposed role in the adaptive response of *B. abortus* to the intracellular environment.

### 2.5. BAB2_0534 Contributes to In Vivo Virulence and Promotes Melanin-Like Deposition in Infected Tissues

To determine the contribution of *BAB2_0534* to *in vivo* persistence, bacterial loads in the spleens of BALB/c mice were quantified. The Δ*BAB2_0534* mutant exhibited impaired persistence, with a significant reduction in splenic bacterial burden at four weeks post-infection (4.00 ± 0.07 log_10_ CFU/spleen) compared with two weeks post-infection (6.28 ± 0.39 log_10_ CFU/spleen; *p* < 0.05). In contrast, the wild-type strain showed only a modest reduction in bacterial burden over the same period, maintaining higher splenic colonization levels. Complementation with pBV1-*BAB2_0534* restored colonization capacity to levels comparable to the wild-type strain (Table 2).

**Figure 9:**
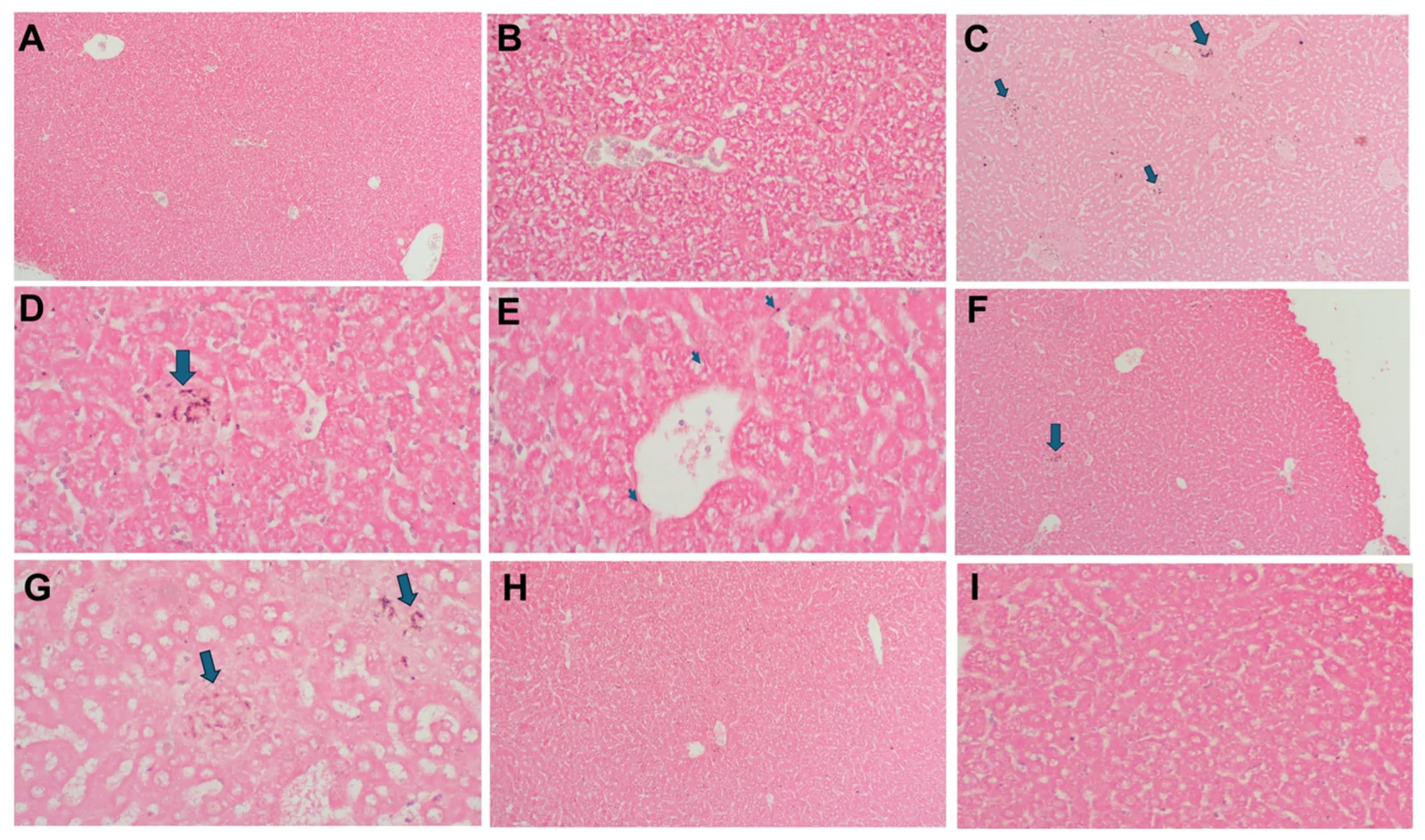
Deletion of *BAB2_0534* prevents detectable in vivo melanin-like pigment deposition and attenuates hepatic pathology in infected mice. Representative histological sections of liver tissues collected from BALB/c mice four weeks after infection. (**A**,**B**) Uninfected control mice showing preserved hepatic architecture without inflammatory lesions or pigment deposition (H&E staining). (**C**–**E**) Mice infected with wild-type *B. abortus* 2308 exhibiting histopathological alterations, including cellular stress, inflammatory infiltrates, and accumulation of a dark, melanin-like argentaffin pigment (arrows), predominantly observed in perivascular regions (Fontana–Masson staining). (**F**,**G**) Mice infected with the complemented strain Δ*BAB2_0534*(pVB1-*BAB2_0534*) displaying comparable but less pronounced histopathological changes and pigment deposition (arrows) (Fontana–Masson staining). (**H**,**I**) Mice infected with the Δ*BAB2_0534* mutant showing reduced hepatic lesions and absence of detectable melanin-like pigment deposits in the examined fields (Fontana–Masson staining). Images are presented at different magnifications to illustrate overall tissue organization (left panels) and cellular-level features (middle and right panels).

Histological analyses were subsequently performed on liver, brain, spleen, kidney, and testis tissues from infected mice. Because hemosiderin and lipofuscin may interfere with melanin detection, melanin-like pigment deposition was primarily evaluated in liver sections using the Fontana–Masson method. H&E staining confirmed the absence of histological alterations in healthy control mice (Figure 9A,B). In contrast, livers from mice infected with wild-type *B. abortus* 2308 exhibited histological changes associated with infection, including cellular stress, bacterial infiltration (Figure 9C–E), and prominent melanin-like pigment deposits, predominantly observed in perivascular regions. Similar, although less extensive, alterations and pigment deposition were detected in mice infected with the complemented strain (Figure 9F,G). Importantly, no melanin-like pigment deposition was detected in liver sections from mice infected with the Δ*BAB2_0534* mutant (Figure 9G,H). Histopathological analysis further revealed differences in lesion severity among experimental groups (Table S1).

## 3. Discussion

Multicopper oxidases (MCOs) are widely distributed among bacteria and are best known for their roles in copper homeostasis and detoxification, including the oxidation of the highly reactive Cu(I) ion to the less toxic Cu (II) state, thereby contributing to protection against copper-mediated toxicity [30,31,32]. More recent studies indicate that these enzymes participate in broader physiological processes, including oxidative stress adaptation, iron metabolism, and bacterial fitness during host infection [33,34]. In this context, our results suggest that *BAB2_0534* encodes a protein predicted to belong to the multicopper oxidase family whose biological role may extend beyond copper homeostasis. The combined genomic, structural, genetic, and infection data are consistent with the hypothesis that this protein contributes to bacterial adaptation during intracellular infection, although its precise enzymatic activity remains to be experimentally confirmed.

Comparative genomic analyses showed that *BAB2_0534* is highly conserved among *Brucella* species and retains the canonical copper-binding motifs characteristic of bacterial multicopper oxidases. Sequence analysis further identified *BAB2_0534* as the *B. abortus* ortholog of the *Brucella* multicopper oxidase BmcO, previously characterized in *B. melitensis* (BMEII0580). Wu et al. (2015) [27] demonstrated that BmcO exhibits oxidase and ferroxidase activities and contributes to bacterial copper tolerance in vitro. Our findings suggest that *BAB2_0534* may have biological functions extending beyond the copper tolerance previously described for BmcO. The phenotypic alterations observed following *BAB2_0534* deletion further support a role for *BAB2_0534* in bacterial adaptation during host–pathogen interactions.

The genomic context of *BAB2_0534* provides additional support for a broader role in stress adaptation. Neighboring genes are predicted to encode proteins involved in redox metabolism, metal transport, and oxidative stress responses, suggesting that *BAB2_0534* may be part of a coordinated adaptive locus. Similar genomic organization has been described for bacterial copper-responsive systems, where multicopper oxidases act together with copper-export ATPases, periplasmic chaperones, and antioxidant enzymes to preserve cellular homeostasis under oxidative conditions [35,36]. Although transcriptional regulation of this locus remains unknown, its genomic organization supports the possibility of coordinated expression during environmental or intracellular stress.

One of the most interesting findings of this study was the association between *BAB2_0534* and melanin-like pigment production. Laccase-mediated melanization is a well-established virulence determinant in fungal pathogens such as *Cryptococcus neoformans*, where melanin contributes to resistance against oxidative stress and phagocyte-mediated killing [37,38,39]. Although comparatively less studied, bacterial melanins have also been proposed to protect microorganisms against reactive oxygen species (ROS), toxic metals, and other environmental stresses [40,41,42]. Under our experimental conditions, deletion of *BAB2_0534* abolished L-DOPA-dependent pigment formation, whereas complementation restored the phenotype. Together with the FTIR analyses, these findings support a role for *BAB2_0534* in phenolic substrates oxidation and melanin-like pigment formation, consistent with the predicted laccase-like multicopper oxidase properties of the encoded protein. However, biochemical characterization of the purified protein will be required to directly establish its catalytic activity and substrate specificity.

The potential biological relevance of this pigment phenotype becomes particularly apparent in the context of intracellular infection. Macrophages restrict bacterial survival through the production of reactive oxygen species, generating a highly oxidative environment that represents an important challenge for *Brucella* persistence [43,44]. Successful intracellular survival relies on multiple, functionally interconnected antioxidant and metal homeostasis systems—including catalases, superoxide dismutases, and metal homeostasis proteins—rather than on a single protective mechanism [12,15,45]. Melanin has been proposed to contribute to microbial stress resistance through ROS scavenging, metal chelation, and free-radical stabilization [24,46]. Consistent with this possibility, the loss of *BAB2_0534* was associated with both increased intracellular ROS accumulation and impaired bacterial survival within macrophages. Although these findings do not establish a direct antioxidant function for the pigment produced by *B. abortus*, they support the hypothesis that *BAB2_0534*-dependent pigment formation may contribute to bacterial adaptation to oxidative stress during intracellular infection.

The reduction in intracellular bacterial survival observed for the mutant strain is compatible with this interpretation. Oxidative stress represents one of the principal selective pressures encountered by *Brucella* following phagocytosis, and defects in bacterial antioxidant systems can impair intracellular replication and persistence [6,43,47,48]. Although the present study does not establish whether *BAB2_0534* directly detoxifies reactive oxygen intermediates, its predicted multicopper oxidase function, together with the increased ROS accumulation and reduced bacterial survival observed in the mutant, supports the hypothesis that *BAB2_0534* contributes to the broader redox homeostasis network that enables *Brucella* to adapt to the intracellular environment.

The metabolic and immunological profiles observed in macrophages further highlight the attenuated phenotype of the Δ*BAB2_0534* mutant. Successful intracellular pathogens, including *Brucella*, actively manipulate host cell metabolism by inducing aerobic glycolysis and increased lactate production to generate a permissive replicative niche [49,50,51,52]. Concurrently, *Brucella* exploits immunoregulatory cytokines, such as IL-10, to limit macrophage activation and promote chronic infection, while suppressing antimicrobial TNF-α responses [53,54]. However, macrophages infected with the Δ*BAB2_0534* mutant failed to exhibit this characteristic increase in lactate and instead mounted a robust pro-inflammatory response, marked by increased TNF-α and impaired IL-10 secretion. Rather than indicating a direct role for *BAB2_0534* in host glycolytic or cytokine regulation, these alterations may reflect the impaired ability of the ROS-stressed mutant to establish the intracellular conditions required for effective persistence and modulation of host–cell responses.

These in vitro observations were further supported by the in vivo infection model. Mice infected with the Δ*BAB2_0534* mutant exhibited significantly lower splenic bacterial burdens than those infected with the wild-type or complemented strains, indicating impaired bacterial persistence during chronic infection. Since long-term colonization of reticuloendothelial organs is a hallmark of *Brucella* pathogenesis, reduced splenic colonization is commonly associated with attenuation of virulence [55,56,57]. Although additional virulence determinants undoubtedly contribute to this phenotype, these findings suggest that *BAB2_0534* contributes to the adaptive mechanisms that enable *Brucella* to establish and maintain intracellular persistence in vivo.

Histopathological analyses provided further evidence for the in vivo relevance of *BAB2_0534*-associated pigment production. The detection of Fontana–Masson-positive pigment in tissues from animals infected with the wild-type strain, together with its absence or marked reduction in animals infected with the Δ*BAB2_0534* mutant, is consistent with the melanization phenotype observed in vitro. Nevertheless, Fontana–Masson staining alone cannot unequivocally distinguish melanin from other argentaffin pigments [58]. Complementary approaches, including electron paramagnetic resonance, Raman spectroscopy, or mass spectrometry, would therefore be valuable for confirming the chemical identity of the pigment [59,60]. Thus, while the histological findings support the presence of melanin-like material in infected tissues, additional biochemical characterization will be required to establish its molecular composition.

Collectively, the results presented here support a working model in which *BAB2_0534* contributes to bacterial adaptation through interconnected processes involving its predicted multicopper oxidase function, phenolic substrate oxidation, oxidative stress tolerance, interaction with host antimicrobial defenses, and intracellular persistence. Rather than functioning as an isolated virulence determinant, *BAB2_0534* may represent one component of a broader adaptive network that enables *B. abortus* to withstand the physicochemical and antimicrobial constraints imposed by the intracellular environment. This concept is consistent with the increasingly recognized view that bacterial virulence and persistence emerge from coordinated physiological responses rather than from the activity of individual genes alone [61,62].

From a translational perspective, these findings open several avenues for future investigation. Biochemical characterization of the purified enzyme, determination of its catalytic properties, transcriptomic analyses under oxidative and copper stress, targeted metabolomic profiling during macrophage infection, and identification of the regulatory network controlling *BAB2_0534* expression will be important to define its precise biological role. Likewise, determining whether inhibition of multicopper oxidase activity increases bacterial susceptibility to host antimicrobial mechanisms could identify potential therapeutic vulnerabilities. Finally, because melanin biosynthesis has emerged as a mechanism contributing to stress resistance and persistence in diverse microbial pathogens, determining whether a similar strategy operates during *B. abortus* infection could broaden current concepts of bacterial adaptation to the intracellular environment and reveal previously unrecognized targets for antimicrobial intervention. Although the BALB/c mouse model provides a useful experimental system for investigating the contribution of *BAB2_0534* to intracellular survival and persistence of *B. abortus*, the generalizability of these findings to the natural bovine host and to other host species remains to be established. Further studies in appropriate host models will be required to determine whether the mechanisms identified in mice are conserved during natural infection.

## 4. Materials and Methods

### 4.1. Animals and Ethics Statement

Healthy, ten-week-old, specific pathogen-free (SPF) female BALB/c mice were obtained from the Institute of Public Health (Santiago, Chile) and housed in the animal facilities of the Laboratory of Molecular Immunology, Department of Microbiology, Faculty of Biological Sciences, Universidad de Concepción (Concepción, Chile). Upon arrival, animals were randomly assigned to four experimental groups and acclimatized for three weeks before the start of the experimental procedures. The four groups comprised a healthy control group and groups infected with wild-type *B. abortus* 2308, Δ*BAB2_0534* mutant, or complemented Δ*BAB2_0534* strains. A total of 40 animals were used, with 10 mice allocated to each experimental group. Within each group, five mice were euthanized at 2 weeks post-infection and five at 4 weeks post-infection. No computer-generated randomization sequence was used, and blinding was not performed during animal allocation, experimental procedures, outcome assessment, or data analysis. Animals were maintained under controlled environmental conditions at an ambient temperature of 20–26 °C, relative humidity of 30–70%, and a 12 h light/12 h dark cycle. Mice were housed in ventilated polycarbonate cages, with five adult animals per cage, and had free access to standard laboratory chow and drinking water ad libitum. Nesting material was provided as environmental enrichment throughout the experimental period.

BALB/c mice were selected as an inbred strain because its relatively homogeneous genetic background helps reduce inter-animal variability and facilitates the evaluation of biological differences associated with bacterial genotype. Sample size was determined using the resource equation method described by Charan and Kantharia [63]. With 20 animals distributed across four experimental groups, the resulting E value was 16 (E = 20 − 4), which falls within the recommended range of 10–20. resulting in an E value of 16 (20 animals − 4 experimental groups), which falls within the recommended range of 10–20. This approach was used to ensure adequate biological replication while adhering to the principle of minimizing animal use. All animal procedures were conducted in accordance with institutional guidelines for the care and use of laboratory animals and were approved by the Institutional Committee for Bioethics and Biosafety of the Universidad de Concepción in May 2023 (Approval No. 1466-2023). All procedures were performed with the aim of minimizing animal stress, discomfort, and suffering. At the designated experimental endpoints, mice were euthanized by cervical dislocation.

### 4.2. Cell Lines

Murine RAW 264.7 macrophages and human cervical epithelial HeLa cells were obtained from the American Type Culture Collection (ATCC, Manassas, VA, USA). Cells were maintained in Dulbecco’s Modified Eagle Medium (DMEM; Thermo Fisher Scientific, Waltham, MA, USA) supplemented with 10% heat-inactivated fetal bovine serum (FBS; Gibco BRL, Grand Island, NY, USA) and 1% antibiotic–antimycotic solution containing 100 U/mL penicillin, 100 μg/mL streptomycin, and 0.25 μg/mL amphotericin B (Sigma-Aldrich, St. Louis, MO, USA). Cultures were incubated at 37 °C in a humidified atmosphere containing 5% CO_2_. Cells were routinely tested and confirmed to be free of mycoplasma contamination.

### 4.3. Bacterial Strains and Culture Conditions

The bacterial strains used in this study are summarized in Table S2. *E. coli* K-12 strains were routinely cultured in Luria–Bertani (LB) broth, whereas *B. abortus* strains were grown in Brucella Broth (Becton Dickinson, Sparks, MD, USA). Unless otherwise indicated, *B. abortus* cultures were incubated at 37 °C for 48–72 h under 5% CO_2_ microaerophilic conditions. When required, media were supplemented with kanamycin (50 μg/mL), ampicillin (100 μg/mL), or chloramphenicol (30 μg/mL), as previously described [64].

### 4.4. Bioinformatic Analysis of BAB2_0534

The *BAB2_0534* open reading frame (ORF) was identified in the *B. abortus* 2308 genome and analyzed to investigate its genomic organization, evolutionary conservation, and predicted structural features. The open reading frame encoding *BAB2_0534* was identified in the *B. abortus* 2308 genome. To confirm gene annotation and evaluate the presence of additional multicopper oxidase homologs, the complete genome sequence was retrieved from GenBank (GCF_000054005.1) and locally reannotated using Prokka [65]. The *BAB2_0534* locus was subsequently analyzed for evolutionary conservation and predicted structural features. Functional associations between *BAB2_0534* and neighboring loci were explored using the STRING database, with emphasis on gene neighborhood, gene co-occurrence, and other genomic-context evidence [66]. Putative operon organization was predicted using Operon-mapper [67]. Gene organization and intergenic distances were visualized with Unipro UGENE v1.25.0 [68] following multiple sequence alignment generated using T-Coffee with default parameters [69].

To evaluate the evolutionary conservation of *BAB2_0534*, phylogenetic analysis was performed using the maximum-likelihood algorithm implemented in RAxML v8.2.12, with the GAMMA model of rate heterogeneity and 1000 bootstrap replicates to assess branch support [70]. The resulting maximum-likelihood phylogenetic tree was visualized and graphically edited using FigTree v1.4.4 [71].

The *BAB2_0534*-encoded protein was further characterized using sequence- and structure-based bioinformatic analyses. The amino acid sequence and functional annotation were retrieved from UniProt (accession Q2YKW0). Physicochemical properties, including molecular weight, theoretical isoelectric point (pI), instability index, aliphatic index, and grand average of hydropathicity (GRAVY), were calculated using the ProtParam tool available through the ExPASy server [72]. The three-dimensional structure of *BAB2_0534* was predicted using AlphaFold 3 [73] and subsequently refined using GalaxyRefine [74]. The structural model was evaluated using complementary validation approaches. Backbone stereochemistry was assessed by Ramachandran plot analysis using PROCHECK [75], overall structural quality was evaluated using ProSA-web [76], and all-atom stereochemical quality, including side-chain rotamer geometry and atomic clashes, was assessed using MolProbity [77].

To assess structural similarity with a characterized multicopper oxidase, the refined *BAB2_0534* model was structurally aligned with the crystal structure of *E. coli* CueO (PDB ID: 1KV7; 1.4 Å resolution), a well-characterized multicopper oxidase involved in copper homeostasis [78]. Structural superposition was performed using PyMOL v1.2r3pre, and the root mean square deviation (RMSD) was calculated to quantify structural similarity between the *BAB2_0534* model and CueO.

### 4.5. Construction of the ΔBAB2_0534 Mutant and Complemented Strain

A *BAB2_0534* deletion mutant (Δ*BAB2_0534*) was generated in *B. abortus* 2308 using a modified λ-Red recombineering system as previously described [79]. Briefly, electrocompetent *B. abortus* 2308 cells were transformed with 200 ng of plasmid pSIM7 [80] and cultured in brucella broth supplemented with 30 μg/mL chloramphenicol at 30 °C for 72 h. Expression of the λ-Red recombinases was induced by incubation at 42 °C for 30 min [81]. A kanamycin resistance cassette (Km^R^), amplified from plasmid pKD4 using the primers listed in Table S2, was introduced by homologous recombination following electroporation. Recombinant clones were selected on Brucella agar supplemented with 50 μg/mL kanamycin and screened by PCR using primers flanking the *BAB2_0534* locus (Table S2). For genetic complementation, the complete *BAB2_0534* coding sequence was amplified from *B. abortus* 2308 genomic DNA using primers incorporating NdeI and BamHI restriction sites (Table S3). The amplified fragment was cloned into plasmid pVB1 to generate pVB1-*BAB2_0534*, which was introduced into the Δ*BAB2_0534* mutant by electroporation, yielding the complemented strain Δ*BAB2_0534* (pVB1-*BAB2_0534*) as previously described [64].

### 4.6. Growth Characterization

To determine whether deletion of the *BAB2_0534* gene affected bacterial growth, growth curves of *B. abortus* 2308 Δ*BAB2_0534*, the complemented strain (*B. abortus* Δ*BAB2_0534*; pVB1-*BAB2_0534*) and wild-type *B. abortus* 2308 were compared. A 100 μL aliquot from each culture was inoculated into Brucella broth and incubated at 37 °C with shaking (150 rpm). Samples were taken every 4 h for 144 h, and optical density was measured at 600 nm. In parallel, 10 μL aliquots were plated in Brucella agar to determine colony-forming unit (CFU/μL), correlating absorbance with CFU counts.

### 4.7. In Vitro Melanin-Like Pigment Production, Purification, and FTIR Characterization

To evaluate melanin-like pigment production, *B. abortus* 2308 wild-type, Δ*BAB2_0534*, and complemented strains were cultured in Brucella broth or on Brucella agar supplemented with increasing concentrations of the phenolic substrates L-3,4-Dihydroxy-L-phenylalanine (L-DOPA) and homogentisic acid (HA), either individually or in combination. Pigment production was monitored during incubation by visual assessment of diffusible pigment formation in liquid cultures and on agar plates.

For pigment purification, bacterial cultures supplemented with L-DOPA were processed using a modified protocol for microbial melanin extraction. Following pigment development, cultures were filtered and acidified to pH 1.5 with hydrochloric acid before centrifugation [20,23,37]. The resulting pigment-containing pellet was repeatedly washed with ethanol, resuspended in sodium hydroxide, and subjected to successive purification cycles until a dark-brown pigment preparation was obtained, following previously described procedures with minor modifications [40]. Purified pigments were dehydrated prior to spectroscopic analysis.

The structural and functional group characteristics of the recovered pigments were evaluated by Fourier-transform infrared spectroscopy (FTIR). Purified pigment extracts obtained from cultures supplemented with 10–100 nM L-DOPA, homogentisic acid (HA), or a combination of both substrates were analyzed in parallel with a commercial eumelanin standard (M2649, Sigma-Aldrich, St. Louis, MO, USA). For FTIR analysis, 0.8 mg of each dried pigment sample was thoroughly mixed with KBr, compressed into transparent pellets, and analyzed using a Nicolet Nexus FTIR spectrophotometer (Thermo Nicolet, Madison, WI, USA) equipped with a DTGS-KBr detector. Spectra were recorded over the range of 4000–400 cm^−1^ and compared to identify characteristic absorption bands associated with melanin-related functional groups-including hydroxyl, carbonyl, and carboxyl moieties, as well as aromatic structure-and to assess the degree of spectral similarity between the bacterial pigments and the eumelanin reference [60].

### 4.8. Intracellular Infection Assay

#### 4.8.1. Intracellular Survival

The effect of *BAB2_0534* gene deletion on intracellular survival was evaluated in vitro using professional (RAW264.7 macrophages) and non-professional (HeLa epithelial cells) phagocytic cell lines. *B. abortus* strains grown for 48 h were adjusted to 10^7^ CFUml^−1^ in DMEM (10% FBS, 2 mM glutamine, antibiotic-free). Bacteria were co-cultured with HeLa or RAW264.7 cells at a multiplicity of infection (MOI) of 500:1 and 50:1, respectively, and incubated for 1 h at 37 °C in 5% CO_2_. The monolayer was washed with PBS and incubated for 60 min in DMEM containing 50 μg ml^−1^ gentamicin and 100 μg ml^−1^ streptomycin to eliminate extracellular bacterial. At 4, 24, 48, and 72 h post-infection, cells were washed and lysed with 0.1% Triton X-100, and serial dilutions were plated on brucella agar to determine CFU/ml [82].

#### 4.8.2. Reactive Oxygen Species (ROS) Production

RAW 264.7 macrophages (5 × 10^5^ cells/well) were seeded in 24-well plates and infected at an MOI of 50:1 for 1 h. After washing with gentamicin, cells were incubated for 24 h at 37 °C in 5% CO_2_. ROS detection was performed using the CellROX kit (Thermo Fisher Scientific, Waltham, MA, USA) according to the manufacturer’s protocol. Negative controls were pretreated with N-acetyl-cysteine (NAC, 200–5000 µM, 1 h), and positive controls with tert-butyl hydroperoxide (TBHP, 100–400 µM, 30–60 min). Cells were stained with Zombie Violet Viability dye (Thermo Fisher Scientifics, Waltham, MA, USA) [83] and labeled with APC/Cy7-conjugated anti-mouse CD11b antibody (1:1000, PBS + 5% FBS). Samples were analyzed on a BD LSR Fortessa™ X-20 flow cytometer using FlowJo software (v10.8, BD Biosciences, Franklin Lakes, NJ, USA) [84].

#### 4.8.3. Lactate Quantification

To assess metabolic changes, intracellular lactate levels in RAW 264.7 cells infected with the different strains were measured using the Lactate Assay Kit (MAK064, Sigma-Aldrich, St. Louis, MO, USA). A lactate standard curve (0, 2, 4, 6, 8, and 10 nmol/well) was prepared in a 96-well plate. After infection, cells were washed, lysed, and centrifuged (200× *g* for 10 min at 4 °C) to remove insoluble material. Supernatants were mixed with Master Reaction Mix and incubated for 30 min at room temperature in the dark. Absorbance was measured at 570 nm, and lactate concentrations were calculated from the standard curve [85].

#### 4.8.4. Cytokine Determination

To analyze cytokine production RAW 264.7 cells (1 × 10^6^ cells/well) were infected with the different *Brucella* strains at a 1:20 MOI for 1 h in antibiotic-free DMEM. After washing with medium containing 50µg/mL gentamicin, cells were incubated for 2, 6 and 12 h. Supernatants were collected, and IL-10, TNF-α levels were determined by ELISA (Invitrogen, Thermo Fisher Scientific, Waltham, MA, USA). All assays were performed in triplicate [86].

### 4.9. Mouse Infection Assay

#### 4.9.1. Bacterial Colonization

To evaluate bacterial colonization in vivo, mice were inoculated intraperitoneally with 1 × 10^5^ CFU of each *B. abortus* strain suspended in 0.1 mL of sterile phosphate-buffered saline (PBS). At 2 and 4 weeks post-infection, mice were euthanized, and spleens were aseptically removed, weighed, and homogenized in sterile PBS. Homogenates were serially diluted, and aliquots of each dilution were plated onto Columbia agar supplemented with 5% sheep blood (bioMérieux, Marcy-l’Étoile, France). Following incubation at 37 °C for 72 h, bacterial burdens were determined by counting colony-forming units (CFU) and expressed as CFU per spleen [87].

#### 4.9.2. Histopathology

For histopathological analysis, the same BALB/c mice described in Section 4.9.1 were used, and they were sacrificed 4 and 8 weeks after infection. Tissue samples, including the spleen, liver, brain, and testis, were collected and fixed in 10% neutral buffered formalin (NBF). These organs were selected because they represent major target tissues during systemic brucellosis. The spleen and liver are the primary sites of bacterial replication and chronic persistence within the reticuloendothelial system, whereas the brain and testis were included to evaluate potential dissemination to immune-privileged organs frequently associated with chronic complications such as neurobrucellosis and *Brucella*-induced orchitis [57,87]. In addition, these tissues provide an appropriate framework to assess the possible accumulation of melanin-like pigments during chronic infection. Paraffin-embedded tissue sections were processed for histological evaluation. Liver lesions were assessed using the NAFLD Activity Score (NAS) based on steatosis, lobular inflammation, and hepatocellular ballooning degeneration [88,89]. To evaluate pigment deposition, tissue sections were stained using the Fontana–Masson method, which specifically detects argentaffin and melanin-like pigments, together with Prussian Blue staining to discriminate melanin deposits from ferric iron (hemosiderin)-containing pigments [58]. Histological analyses were performed Laboratory of Pathological Anatomy and Descriptive Pathophysiology, Faculty of Medicine, Universidad de Concepción.

### 4.10. Statistical Analysis

Statistical analyses were performed using GraphPad Prism (version 9.0; GraphPad Software, San Diego, CA, USA). Data normality was assessed using the Shapiro–Wilk test, and homogeneity of variances was evaluated before parametric analyses. Comparisons involving a single independent variable were performed using one-way analysis of variance (ANOVA), whereas datasets including two independent variables (bacterial strain and time) were analyzed by two-way ANOVA. When significant differences were detected, Tukey’s multiple-comparison test was applied for post hoc analyses. A *p* value < 0.05 was considered statistically significant. Unless otherwise indicated, all experiments were performed using three independent biological replicates with technical replicates, and data are presented as mean ± standard deviation (SD). The number of independent biological replicates (*n*) and the corresponding measure of variability are indicated in the respective figure legends or tables. Effect sizes and their confidence intervals were not calculated.

## 5. Conclusions

This study characterizes *BAB2_0534* as a previously uncharacterized protein predicted to belong to the multicopper oxidase family and shows that it contributes to the adaptive capacity of *B. abortus*. Our findings demonstrate that *BAB2_0534* promotes the oxidation of phenolic substrates and the production of a eumelanin-like pigment, as supported by spectroscopic characterization and comparison with a eumelanin reference. Loss of *BAB2_0534* was associated with increased susceptibility to oxidative stress and impaired intracellular survival in macrophages, indicating that BAB2 contributes to bacterial adaptation to host antimicrobial conditions. Importantly, the Δ*BAB2_0534* mutant displayed significantly reduced splenic bacterial burdens during infection, providing evidence that *BAB2_0534* contributes to bacterial persistence *in vivo*. The reduced detection of Fontana–Masson-positive pigment in tissues infected with the mutant further supports the relevance of *BAB2_0534*-associated pigment production during infection. Collectively, these results support a model in which *BAB2_0534*, a protein with predicted multicopper oxidase features, and its associated eumelanin-like pigment production contribute to oxidative stress resistance, intracellular adaptation, and persistence of *B. abortus*. Further biochemical and molecular studies will be required to define the enzymatic mechanism and regulatory pathways underlying this adaptive phenotype.

## Figures and Tables

**Table 1:** Description according to the genomic annotation of sequences BAB2_0530 to BAB2_0535.

Sequence	Protein	Position in Genome	Size (Bp)
*BAB2_0530* RC	Hydrogen peroxide-inducible genes activator	143,86–44,391	906
*BAB2_0531*	Alkyl hydroperoxide reductase C	144,539–145,039	555
*BAB2_0532*	Alkyl hydroperoxide reductase AhpD	145,173–145,700	528
*BAB2_0533* RC	Inner membrane metabolite transport protein YhjE	145,778–147,058	1281
*BAB2_0534*	Copper oxidase CueO	147,476–149,371	1896
*BAB2_0535*	Superoxide dismutase Cu/Zn SOD	149,644–150,165	522

RC: Reverse complementary of the original sequence.

**Table 2:** Colonization of the spleen of BALB/c mice with *B. abortus* 2308, *B. abortus* 2308 Δ*BAB2_0534* mutant, and complemented *B. abortus* 2308 Δ*BAB2_0534* (pVB1-*BAB2_0534*).

Strains of *B. abortus*	Two Weeks After Infection	Four Weeks After Infection
*B. abortus* 2308	6.78 ± 0.05	5.66 ± 0.05
*B. abortus* Δ*BAB2_0534*	6.28 ± 0.39 *	4.00 ± 0.07 *
*B. abortus* Δ*BAB2_0534* (pVB1-*BAB2_0534*)	6.69 ± 0.04	5.14 ± 0.05

Statistical analyzes were performed comparing mutant strains with wild-type and complemented strains. * *p* < 0.05 showing significantly lower number of CFU/spleen compared to *B. abortus* 2308.

## Data Availability

The datasets used and/or analyzed during the current study are available from the corresponding author on reasonable request.

## Supplementary Materials

The following supporting information can be downloaded at https://www.mdpi.com/article/10.3390/ijms27188105/s1. Reference [89] is also cited in the supplementary materials.

## Abbreviations

The following abbreviations are used in this manuscript:

L-DOPA	L-3,4-dihydroxyphenylalanine
HA	Homogentisic acid
FTIR	Fourier transform infrared spectroscopy
MCOs	Multicopper oxidases
