# Supplementary material for: A Putative Multicopper Oxidase Encoded by BAB2_0534 Promotes Melanin-Like Pigment Production and Intracellular Adaptation in Brucella abortus
Source: Int J Mol Sci. 2026 Sep 11;27(18):8105. doi: 10.3390/ijms27188105 (PMC13607516; doi:10.3390/ijms27188105)
Supplement: Supplementary file 1 [file ijms-27-08105-s001.zip › ijms-4524688-supplementary.pdf]

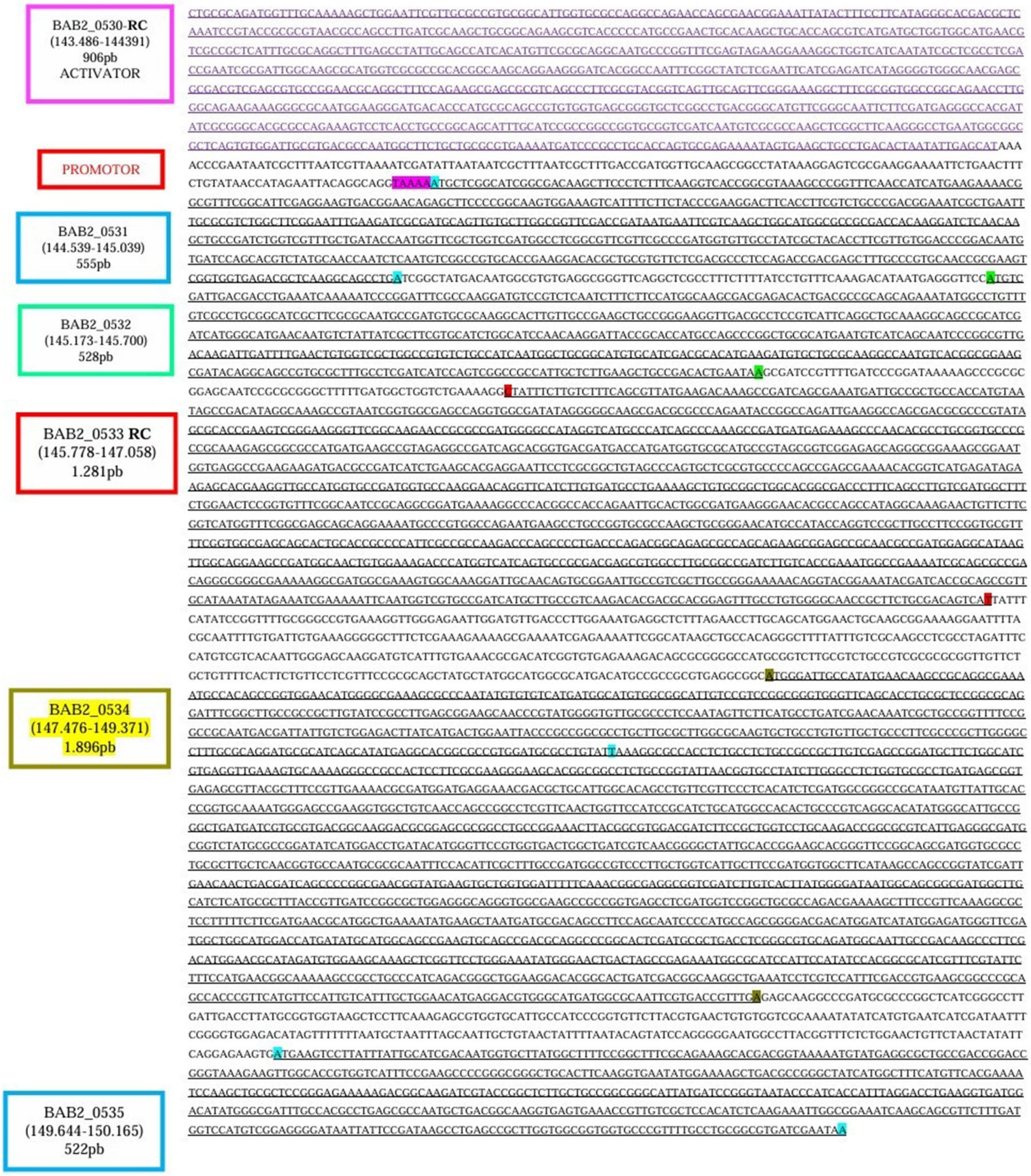

Figure S1: Predicted genomic organization of the *BAB2_0530–BAB2_0535* operon. Operon prediction identified a putative transcriptional unit spanning BAB2_0530 to BAB2_0535. Promoter prediction analysis identified a putative promoter region located between BAB2_0530 and BAB2_0531, suggesting that BAB2_0531–BAB2_0535 may be co-transcribed. The genomic organization and predicted promoter are shown.

Table S1: NAFLD Activity Score (NAS) criteria used for histopathological evaluation of liver tissue

**Strain *Brucella***	**Histological findings with hematoxylin-eosin technique**	**Histological findings with Fontana Masson technique (Liver)**	**Histological classification with NAFLD activity score (NAS) (Keliner et al., 2005)**
B. abortus 2308 Δ*BAB2_0534*	Brain, Cerebellum, Spleen, Kidney: No lesions.<br>Liver: Lymphocytic inflammation around the central hepatic venule in zone 3 with 2-4 foci per 10x magnification, without visible granules.	No pigments are observed in zone 3.	3/1/0<br>Value Score 4<br>Undetermined
Brucella abortus Δ*BAB2_0534* (pVB1-*BAB2_0534*)	Brain, Cerebellum, Spleen, Kidney: No lesions.<br>Liver: Basophilic intracytoplasmic granules in the hepatocytes peri-central hepatic venule in zone 3. No inflammation.	Scant accumulation of lipofuscin-like pigment in zone 3, with 1-2 foci per 10x magnification.	2/2/0<br>Value Score 4<br>Undetermined
B. abortus 2038	Brain, Cerebellum, Spleen, Kidney: No lesions.<br>Liver: Basophilic granules in the hepatocytes peri-central hepatic venule and lymphocytic inflammation in zone 3, with >4 foci per 10x magnification.	Accumulation of lipofuscin-like pigment in the liver parenchyma, with >4 foci per 10x magnification.	3/3/2<br>Value Score 8<br>Definite NASH
Uninfected Control Tissue	Brain, Cerebellum, Spleen, Kidney, Liver: No lesions.	No pigments are observed in zone 3.	0/0/0<br>Value Score 0<br>No NASH
All Control tissues (Brain, Cerebellum, Spleen, Kidney in each case) did not present lesions.			

For the damage analysis of the liver tissue samples present in this work, the NAFLD Activity Score (NAS) system was used, which involves the activity scoring system analyzing 14 histological characteristics that were correlated by logistic regression with the diagnosis of NASH. This score includes 3 parameters with greater statistical significance and evaluated semi-quantitatively that are

added to obtain a score of 0-8 points: steatosis (0-3), lobular inflammation (0-3), balloonization (0-2). Correlation between the total NAS score and the overall histological diagnosis of steatohepatitis. NAFLD activity score Histological diagnosis of steatohepatitis. ≥ 5 probable or definite NASH, 3-4 Undetermined, ≤ 2 No NASH [89].

Table S2. Bacteria and plasmids used in this study

STRAIN PLASMID	CARACTERISTICS	REFERENCESS
B. abortus 2308	Wild Strain, soft and virulent	Our Laboratory
B. abortus 2308 Δ*BAB2_0534*	*B. abortus* 2308, eliminated in the ORF *BAB2_0534*	This Work
B. abortus 2308 Δ*BAB2_0534* (pVB1-*BAB2_0534*)	*B. abortus* Δ*BAB2_0534*, that contains the plasmid pVB1-*BAB2_0534*, Am^r, Km^r	This Work
Escherichia coli K-12	*(rK–, mK+) phoA supE44 λ- thi-1 gyrA96 relA1*	Invitrogen
pSIM7/pSIM9	Broad host range cloning vector,Lambda Red Recombinase (λ-Red)	Our Laboratory
pKD4	Secuences Km^r	Our Laboratory
pVB1	Cloning vector for expression of PCR products	Our Laboratory
pVB1-*BAB2_0534*	Recombinant vector encoding the gene *BAB2_0534* of *B. abortus 2308*	This Work

*Am^r - Ampicillin resistance; Km^r - Kanamycin resistance.

Table S3. Sequences of genes and primers used in this work

Name	Secuences	Restriction Enzyme	Size (pb)
BAB2_0534 **Kmr (F)**	GCAAGCCTCGCCTAGATTTCCATGTCGTCA*	NA	
BAB2_0534 **Kmr (R)**	CATATGAATATCCTCCTTAGTTCCTATTCC*	NA	
BAB2_0534 **(F)**	AAA*AAGCTT*GCCACCATGGGATTGCCATATGAACA	†HinD III	
BAB2_0534 **(R)**	TCAAACGGTCACGAATTGCG*GGATCC*TTT	†BamH I	
BAB2_0534	ATGGGATTGCCATATGAACA AGCC…CGCAATTCGTGACCGTTTGA	NA	1605

*The Sequence used to amplify Kmr is underlined. †NdeI and BamHI, restriction endonuclease cleavage sites are underlined. ND, not available; (F), Go ahead; (R), reverse.

References

89. Takahashi, Y. Animal Models of Nonalcoholic Fatty Liver Disease/Nonalcoholic Steatohepatitis. *World J. Gastroenterol.* **2012**, *18*, 2300, doi:10.3748/wjg.v18.i19.2300.
